# CRISPR/Cas9-Based Gene Editing Using Egg Cell-Specific Promoters in Arabidopsis and Soybean

**DOI:** 10.3389/fpls.2020.00800

**Published:** 2020-06-16

**Authors:** Na Zheng, Ting Li, Jaime D. Dittman, Jianbin Su, Riqing Li, Walter Gassmann, Deliang Peng, Steven A. Whitham, Shiming Liu, Bing Yang

**Affiliations:** ^1^State Key Laboratory for Biology of Plant Diseases and Insect Pests, Institute of Plant Protection, Chinese Academy of Agricultural Sciences, Beijing, China; ^2^Division of Plant Sciences, Christopher S. Bond Life Sciences Center, and Interdisciplinary Plant Group, University of Missouri, Columbia, MO, United States; ^3^The Division of Biology and Biological Engineering, California Institute of Technology, Pasadena, CA, United States; ^4^Department of Plant Pathology and Microbiology, Iowa State University, Ames, IA, United States; ^5^Donald Danforth Plant Science Center, St. Louis, MO, United States

**Keywords:** CRISPR/Cas9, gene editing, egg cell-specific promoter, Arabidopsis, *Glycine max*, soybean

## Abstract

CRISPR/Cas9-based systems are efficient genome editing tools in a variety of plant species including soybean. Most of the gene edits in soybean plants are somatic and non-transmissible when Cas9 is expressed under control of constitutive promoters. Tremendous effort, therefore, must be spent to identify the inheritable edits occurring at lower frequencies in plants of successive generations. Here, we report the development and validation of genome editing systems in soybean and Arabidopsis based on Cas9 driven under four different egg-cell specific promoters. A soybean ubiquitin gene promoter driving expression of green fluorescent protein (GFP) is incorporated in the CRISPR/Cas9 constructs for visually selecting transgenic plants and transgene-evicted edited lines. In Arabidopsis, the four systems all produced a collection of mutations in the T2 generation at frequencies ranging from 8.3 to 42.9%, with egg cell-specific promoter AtEC1.2e1.1p being the highest. In soybean, function of the gRNAs and Cas9 expressed under control of the CaMV double 35S promoter (2x35S) in soybean hairy roots was tested prior to making stable transgenic plants. The 2x35S:Cas9 constructs yielded a high somatic mutation frequency in soybean hairy roots. In stable transgenic soybean T1 plants, AtEC1.2e1.1p:Cas9 yielded a mutation rate of 26.8%, while Cas9 expression driven by the other three egg cell-specific promoters did not produce any detected mutations. Furthermore, the mutations were inheritable in the T2 generation. Our study provides CRISPR gene-editing platforms to generate inheritable mutants of Arabidopsis and soybean without the complication of somatic mutagenesis, which can be used to characterize genes of interest in Arabidopsis and soybean.

## Introduction

With the advent of genome editing technologies, such as zinc-finger nucleases (ZFNs), transcription activator-like effector nucleases (TALENs), and clustered regularly interspaced short palindromic repeat (CRISPR) and CRISPR-associated protein (Cas) (CRISPR/Cas) in particular, targeted mutagenesis and precise base changes in genomes of interest can be achieved in ways that were unimaginable 10 years ago (Zhang et al., 2018). These engineered nucleases can generate double-stranded DNA breaks (DSBs) at pre-chosen genomic loci, and repairs to the DSBs *in vivo* lead to site-specific genetic alterations. Two main pathways are used to repair DSBs *in vivo*: error-prone non-homologous end joining (NHEJ) and error-free homology-directed repair (HDR) in the presence of template DNA. The former is the predominant event that introduces insertions/deletions (indels) that range from one to hundreds of base pairs (Voytas, 2013).

The type II CRISPR/Cas9 system from *Streptococcus pyogenes* is the first described and the most popular CRISPR system for genome editing. CRISPR/Cas9 consists of two components, the Cas9 nuclease and a chimeric single guide RNA (gRNA) derived from the fusion of a crRNA (CRISPR RNA) and a trans-activating crRNA preceded by a spacer (or guide) sequence of 18–20 nucleotides complementary to the target DNA (or protospacer). The Cas9 protein cleaves the target DNA to cause DSBs predominantly located 3 bp upstream of the protospacer adjacent motif (PAM) sequence (5′-NGG-3′). Due to the ease of assembly and high frequency of inducing mutations, the CRISPR/Cas9 system is widely used for gene editing in various organisms, including yeast, mouse, fish, human cells, and plant species (Jinek et al., 2012; Cong et al., 2013; Hwang et al., 2013; Jiang et al., 2013; Li et al., 2013; Mali et al., 2013; Nekrasov et al., 2013; Shan et al., 2013).

Soybean (*Glycine max*) is one of the most economically important crops for food, vegetable oil and animal feed. As a paleopolyploid species, gene function studies in soybean are frequently complicated by genetic redundancy, in addition to low frequencies of genetic transformation. Nevertheless, CRISPR/Cas9 genome editing systems have been successfully utilized in soybean since the first demonstrations in 2015. It has been applied to create targeted mutations in hairy roots, somatic embryos and stable transgenic plants (Jacobs et al., 2015; Li et al., 2015, 2019; Michno et al., 2015; Sun et al., 2015; Du et al., 2016; Chilcoat et al., 2017; Cai et al., 2018, 2020; Kanazashi et al., 2018; Al Amin et al., 2019; Bao et al., 2019; Campbell et al., 2019; Cheng et al., 2019; Do et al., 2019; Zhang et al., 2019; Michno et al., 2020; Wang et al., 2020; Wu et al., 2020). Various promoters have been deployed for expression of Cas9. For example, the constitutive cauliflower mosaic virus (CaMV) 35S promoter (35S) is the most used, especially in hairy roots (Jacobs et al., 2015). The soybean SCREAM M4 promoter (pM4) (Bai et al., 2020), parsley ubiquitin promoter (Kanazashi et al., 2018), and translation elongation factor 1 alpha 2 (*EF1A2*) promoter (Li et al., 2015) have also been used to constitutively activate Cas9 expression.

Compared with the constitutive promoters, germline specific promoters for Cas9 expression can improve the frequency and heritability of mutations significantly in Arabidopsis. Expression of Cas9 under control of the *DD45* (egg cell and early embryo), *Yao* (shoot apical and root meristem-active), tomato *Lat52* (pollen) and *EC* (egg cells, embryo) promoters can increase the frequency of heritable edits in the T2 generation. These promoters also reduce the rate of somatic mutations (Wang et al., 2015; Yan et al., 2015; Mao et al., 2016). The lower frequency of chimerism reduces the need to screen large numbers of individual plants and conduct multiple generational analyses to acquire the desired mutants in Arabidopsis. The efficiency of *Agrobacterium*-mediated soybean transformation is very low, labor-intensive and time-consuming, so a high-efficiency CRISPR/Cas9 system based on germline specific promoters may reduce the chimerism and thus, the workload of characterizing edited plants.

In this work, we present easy-to-use binary vector systems with Cas9 driven by egg cell-specific promoters (ECp) for efficient site-specific mutagenesis in Arabidopsis and soybean based on *Agrobacterium*-mediated transformation. In the system, a GFP marker can also be used to identify transgenic and transgene-free plants. We validated the ECp-Cas9 systems and *Agrobacterium*-mediated protocol by targeting two genes each in Arabidopsis and soybean. Our results showed that egg cell-specific promoters can induce mutations of endogenous genes in Arabidopsis and soybean, and multiple, independent mutations can be obtained from the progeny of individual single lines. We confirmed that the continuous presence of the Cas9/gRNA construct in transgenic plants can cause mutagenesis of target genes of interest in subsequent generations.

## Results

### Construction of an ECp-Cas9/gRNA System for Genome Editing in Arabidopsis and Soybean

**FIGURE 1:**
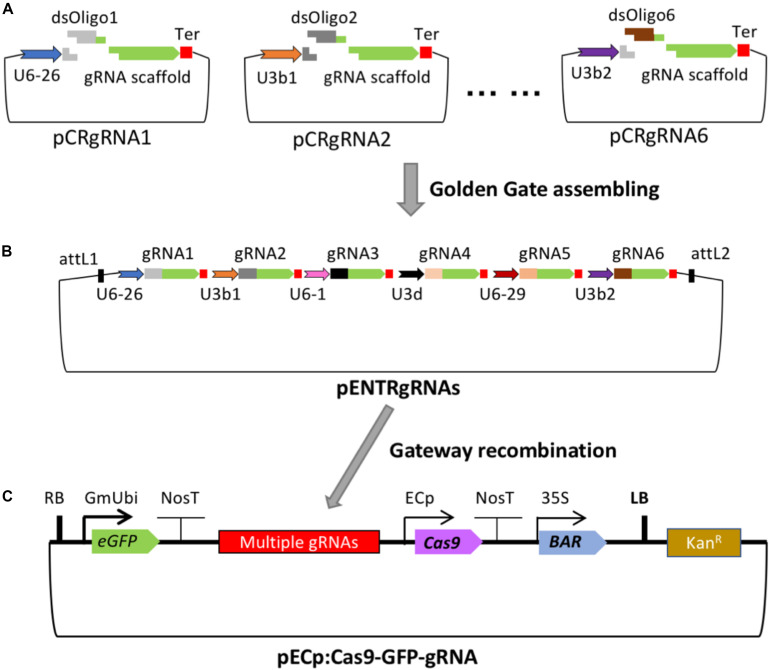
Cloning strategy for adding gRNA modules to the T-DNA vectors carrying Cas9, eGFP, and Bar. **(A)** The pCRgRNA vectors (with two *BsmB*I and two *Bsa*I recognition sites) have different *Arabidopsis* U6 or U3 promoters to drive expression of each gRNA. A common transcription terminator (Ter) follows each gRNA scaffold. Each gRNA vector can be digested with *Bsm*BI for the insertion of double-stranded oligonucleotides as the guide sequence of a specific gRNA (dsOligo#). **(B)** Up to six unique gRNA expression cassettes can be assembled into an intermediate construct, pENTR4-sgRNAs. **(C)** The gRNA module flanked by the *att*L1 and *att*L2 sites is mobilized into the binary vector P1300-ECp-Cas9-GFP-ccdB by Gateway recombination. The resulting Cas9/sgRNA binary construct is used for *Agrobacterium*-mediated transformation. The features of the plasmids are not drawn to scale.

**FIGURE 2:**
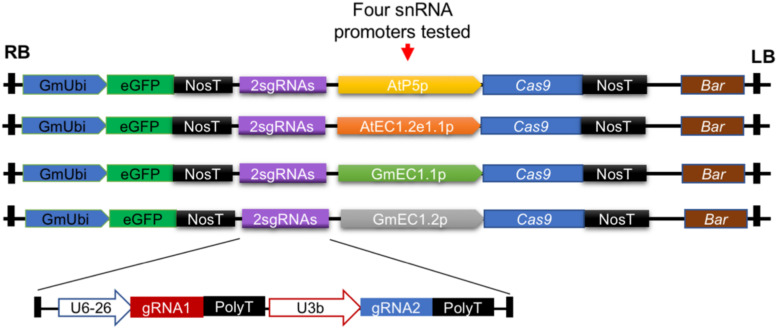
Design of constructs to test ability of four different egg cell-specific promoters to activate expression of Cas9 in Arabidopsis and soybean. In these studies, a gRNA module expressing two different gRNAs (2sgRNAs) targeting unique loci in Arabidopsis and soybean was used. GmUbi, soybean ubiquitin promoter; eGFP, enhanced green fluorescent protein; NosT, nopaline synthase terminator; Bar, bialaphos resistance.

The cloning strategy for building the series of gRNA vectors for insertion into the destination ECp-Cas9/gRNA binary vector suitable for *Agrobacterium*-mediated transformation of Arabidopsis and soybean is shown in Figure 1. The gRNA vectors (pCRgRNA1 to pCRgRNA6) were designed and constructed to generate individual gRNA units each consisting of a small nuclear RNA (snRNA, U6 or U3) promoter and a gRNA followed by a poly-T terminator. The multiple cloning sites between the U6 or U3 and the gRNA scaffold sequence contain two *Bsm*BI sites which facilitate an insertion of a double-stranded DNA fragment with two unique overhangs generated after annealing two complementary oligonucleotides. The inserted sequence in each gRNA vector forms the spacer or guide sequence of a gRNA gene designed specifically to target the genomic locus of interest (Figure 1A). The six gRNA cassettes were designed and constructed such that, after digestion with *Bsa*I, each unit has overhanging ends compatible to the ends of the adjacent units, which are assembled into the intermediate pENTR_ccdB vector using Golden Gate assembly (Supplementary Table S1). In order to have the flexibility of making constructs containing fewer than 6 gRNA units, two additional vectors (pCRgRNA2T and pCRgRNA4T) were designed and constructed to enable 2- and 4-gRNA units (Supplementary Table S1). Therefore, the vectors can be used to construct gRNA modules consisting of 2, 4, or 6 gRNAs (Figure 1B). In this study, we used the 2-gRNA version to target two different genes at once for mutagenesis (Figure 2).

For egg cell-specific expression of Cas9, promoters of four egg cell-specific genes were fused to the Cas9 coding sequence to establish the four CRISPR/Cas9 systems used in this study (Figure 2). This strategy was used to allow us to identify the promoter that worked best for genome editing in both Arabidopsis and soybean. The promoter from the AT1G71470 locus of Arabidopsis, referred to as AtP5p, was used for expression of the Arabidopsis codon-optimized Cas9. Another Arabidopsis promoter, referred to as AtEC1.2e1.1p, was adapted from the fusion of AtEC1.1 and AtEC1.2 *cis*-regulatory elements (Wang et al., 2015). Additionally, two soybean promoters [GmEC1.1p and GmEC1.2p from loci encoding *G. max* egg cell-secreted protein 1.1 (LOC100801164) and *G. max* egg cell-secreted protein 1.2 (LOC102670289), respectively] were identified and used for Cas9 expression. The Cas9 expression plasmids each contained a cassette of the *ccdB* gene flanked by the Gateway recombination sequences *att*L1 and *att*L2 (Supplementary Figure S1). The gRNA modules from pENTR-gRNAs could be mobilized to the individual destination vectors pGW-ECp:Cas9-GmUbi:GFP through Gateway recombination, resulting in a single binary vector pECp:Cas9/gRNA for *Agrobacterium*-mediated plant transformation with the *bar* gene driven by the CaMV 35S promoter as the transformation selection marker (Figures 1C, 2).

The guide sequences of gRNA genes were selected and designed based on the *Arabidopsis thaliana* ecotype Wassilewskija-2 (WS-2) or soybean cultivar Williams 82 genome sequences using the CRISPR Genome Analysis Tool (Brazelton et al., 2015)^1^. The corresponding target genomic regions were PCR-amplified and confirmed by Sanger sequencing prior to gRNA design. All pCRgRNA constructs were confirmed for sequence accuracy at the insertion sites and the flanking regions by Sanger sequencing. The binary plasmids were mobilized into *Agrobacterium* strain GV3101 or EHA105 for transformation of Arabidopsis and soybean, respectively.

**FIGURE 3:**
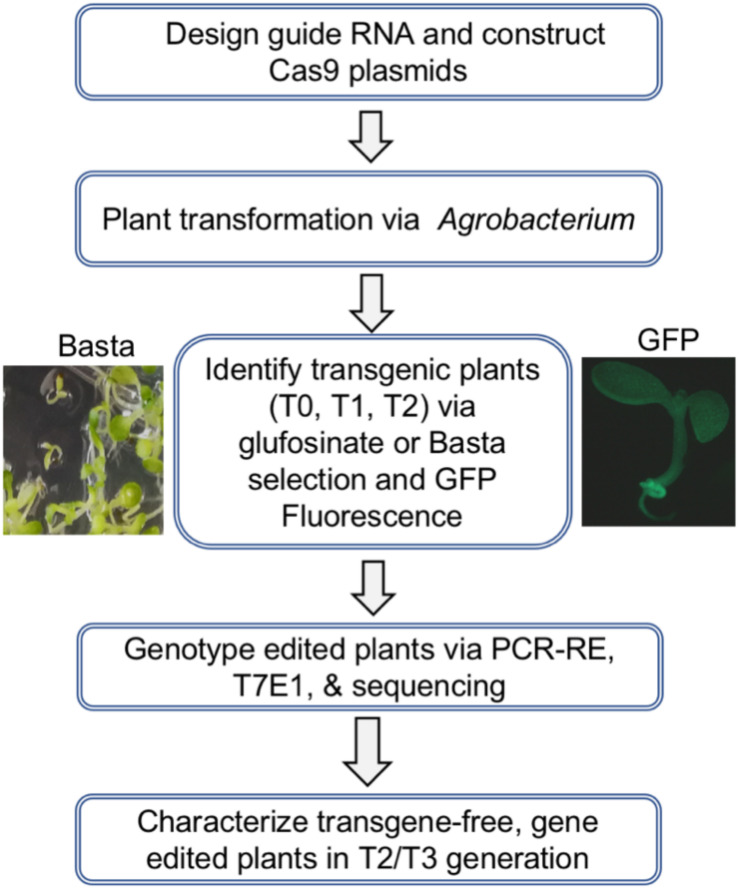
Overall strategy to generate stable transgenic Arabidopsis and soybean lines and identify progeny plants carrying site-specific mutations.

The overall strategy to design and apply a CRISPR/Cas9 construct for targeted mutagenesis in Arabidopsis and soybean is illustrated in Figure 3. The main steps include designing and constructing individual gRNA gene units, assembling the gRNA units into a guide RNA cassette, transferring the gRNAs into ECp:Cas9 vectors, transforming *Agrobacterium*, and performing *Agrobacterium*-mediated plant transformation. Basta resistance and GFP fluorescence were used to screen and select for the transgenic or transgene-free plants of the T0, T1, and T2 generations. The final step involves genotyping edited plants through PCR-amplification of the targeted regions followed by restriction enzyme digestion analysis (PCR-RE) or T7 endonuclease I (T7E1) assay and further confirmation of edits by Sanger sequencing (Figure 3).

### Targeted Mutagenesis of *AtRPS4* and *AtRPS4B* in Arabidopsis

**FIGURE 4:**
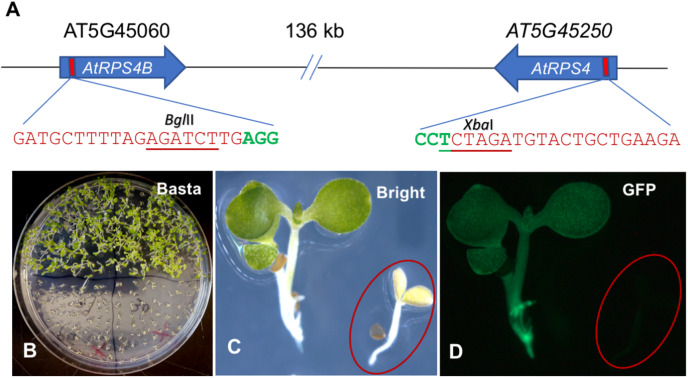
Generation of Arabidopsis plants carrying pECp-Cas9/2sgRNA constructs targeting *AtRPS4* and *AtRPS4B.* **(A)** Schematic representation of *AtRPS4* and *AtRPS4B* and their gRNA target sites. Nucleotides in red and green indicate gRNA target sites and the PAM sequence, respectively. Underlined nucleotides represent the restriction enzyme site in the target gene used to detect mutations. **(B)** Plants grown in MS solid medium with Basta. **(C,D)** Transgenic plants under the bright and fluorescent (GFP) light, respectively. circles indicate the location of a seedling not obviously visible under fluorescent light in **(D)**.

To investigate whether the ECp-Cas9/gRNA systems could induce site-specific mutations, we first tested the ability of each to induce mutations in Arabidopsis. The Arabidopsis genes encoding the TIR-NB-LRR proteins AtRPS4 and AtRPS4B were chosen as the targets. These resistance proteins activate effector-triggered host immunity upon recognizing two bacterial effectors, AvrRps4 from *Pseudomonas syringae* and PopP2 from *Ralstonia solanacearum* (Saucet et al., 2015). The two gRNA genes (gRPS4 and gRPS4B, one gRNA for each target gene) were designed and constructed into one gRNA cassette using pCRgRNA1 and pCRgRNA2T as intermediate cloning vectors. The Cas9 cleavage site (three nucleotides upstream of the NGG PAM) of each target gene overlapped with a restriction enzyme recognition site (*Xba*I for *AtRPS4* and *Bgl*II for *AtRPS4B*), which facilitated genotyping using the PCR-RE approach (Figure 4A).

The same gRNA cassette was mobilized individually into pGW-ECp:Cas9-GmUbi:GFP recipient vectors, resulting in four different ECp:Cas9/gRNA constructs that were individually introduced into Arabidopsis through *Agrobacterium*-mediated transformation using the floral-dipping method. The Bar and GFP markers were used to select the transgene positive plants. Compared with non-transgenic Arabidopsis WS-2 plants, the transgenic plants grew well on the MS solid medium supplemented with Basta and showed strong green fluorescence under fluorescence microscopy (Figures 4B–D).

We first established a way to identify true transgenic plants by combining Basta resistance and GFP fluorescence presence to screen the T1 and T2 generations. Several transgenic T1 plants obtained from four different ECp:Cas9/gRNA constructs were subjected to Basta, GFP fluorescence and Cas9/gRNA-PCR screening (Supplementary Table S2). Compared to Basta screening, the GFP fluorescence was more consistent with the presence of the gRNA genes and Cas9 as further confirmed by the PCR approach (Supplementary Table S2). These results indicated that transgenic plants could be best identified by using the two methods together.

We next examined the extent of gene editing in the T1 populations by PCR-amplifying the relevant genomic regions and digesting the amplicons with the restriction enzymes *Xba*I for *AtRPS4* and *Bgl*II for *AtRPS4B* (PCR-RE approach) followed by Sanger sequencing some of the randomly selected amplicons. For AtEC1.2e1.1p, among 20 transgenic T1 lines selected, 3 lines contained mutations at one target locus (*AtRPS4*) (Supplementary Table S2). Two mutations were heterozygous, while one was bi-allelic. The frequency of mutations for *AtRPS4* was 15%, suggesting that the T-DNA transferred into zygotes of Arabidopsis flowers started to function at an early stage. No mutation was detected at the *AtRPS4B* target locus, suggesting a low efficiency of the guide-RNA for *AtRPS4B*. However, no mutation was detected at the two target loci with the three constructs containing other three ECp driving Cas9 expression. These results indicate that site-specific mutants can be obtained in the T1 generation with one of four egg cell promoters.

**FIGURE 5:**
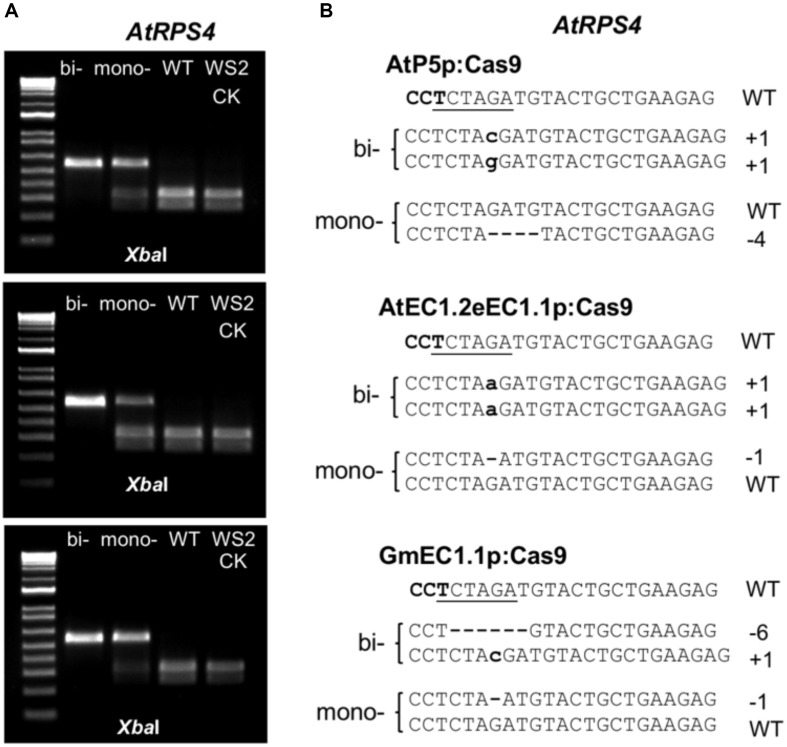
Genotyping Arabidopsis T2 progeny with the PCR-RE and sequencing approaches. **(A)** Gel images of PCR-amplicons from the segregating T2 progeny along with parent WS2 digested with *Xba*I. **(B)** Sequencing results of PCR amplicons derived from the plants as shown in **(A)**. bi-, biallelic mutants; mono-, monoallelic mutants; WT, wild type segregants; WS2 CK, parent control.

Since ECp:Cas9/gRNA should continue to function in egg cells, zygotes and the early stage embryos in the reproductive stage of transgenic plants in the T1 or successive generations, we focused on detecting mutations at the target loci in the T2 generation derived from each CRISPR construct. We counted T2 plants that survived Basta selection in MS medium and with GFP fluorescence and calculated the segregation ratio from individual T1 lines (Supplementary Table S3). The T2 plants from several T1 lines with a 3:1 segregation ratio for each Cas9/gRNA construct were selected for genotyping using the PCR-RE approach (Figure 5). For AtEC1.2e1.1p, the mutation frequencies ranged from 18.2 to 63.6% for *AtRPS4* and from 0 to 18.2% for *AtRPS4B* (Table 1). Genotyping and sequencing analysis of T2 plants showed that AtP5p, GmEC1.1p and GmEC1.2p induced site-specific mutations at 12.8, 25.2, and 0%, respectively, for *AtRPS4*, but induced no mutation at the *AtRPS4B* target locus (Table 1). The AtEC1.2e1.1p:Cas9 construct induced the highest number of mutations at both loci in comparison with the other egg cell-specific promoters. These results demonstrated that AtEC1.2e1.1p was the most efficient promoter compared to AtP5p, GmEC1.1p and GmEC1.2p for gene editing in Arabidopsis.

**TABLE 1:** Efficiency of targeted mutagenesis by Cas9 driven by four different promoters in T2 generation of Arabidopsis.

Promoter for Cas9	T1 Line	*AtRPS4* number of plants	Editing efficiency	*AtRPS4B* number of plants	Editing efficiency	Total T2 plants
AtEC1.2e1.1p	#18	WT(4)/Heter-(3)/Homo-(2)/Bi-(2)	63.6%	WT(9)/Heter-(1)/Bi-(1)	18.2%	11
	#21	WT(4)/Heter-(3)/Bi-(2)	55.6%	WT(9)	0%	9
	#26	WT(8)/Heter-(1)/Bi-(1)	20%	WT(9)/Heter-(1)	10.0%	10
	#42	WT(9)/Bi-(2)	18.2%	WT(10)/Heter-(1)	9.0%	11
	#46	WT(3)/Heter-(1)/Bi-(3)	57.1%	WT(7)	0%	7
AtP5p	#5	WT(15)	0%	WT(15)	0%	15
	#11	WT(8)/Heter-(2)	20.0%	WT(10)	0%	10
	#20	WT(7)/Heter-(3)/Bi-(1)	36.4%	WT(11)	0%	11
	#27	WT(12)	0%	WT(12)	0%	12
	#41	WT(12)/Heter-(1)	7.8%	WT(13)	0%	13
GmEC1.1p	#10	WT(9)/Heter-(3)	25.0%	WT(12)	0%	12
	#36	WT(13)	0%	WT(13)	0%	13
	#42	WT(5)/Heter-(4)/Bi-(3)	63.6%	WT(12)	0%	12
	#52	WT(7)/Heter-(1)	12.5%	WT(8)	0%	8
	#72	WT(9)/Heter-(2)/Bi-(1)	25.0%	WT(12)	0%	12
GmEC1.2p	#2	WT(27)	0%	WT(27)	0%	27
	#3	WT(24)	0%	WT(24)	0%	24
	#4	WT(4)	0%	WT(4)	0%	4
	#5	WT(19)	0%	WT(19)	0%	19

*Hetero-, heterozygous mutants; Bi-, biallelic mutants; WT, wildtype segregants.*

We also determined the exact identities of edits in individual T2 plants from the single T1 lines. Sanger sequencing was performed for seven plants from line #18 of AtEC1.2e1.1p:Cas9 construct, and five plants contained single mutations for *AtRPS4* and two contained double mutations for *AtRPS4* and *AtRPS4B*. A variety of mutations (heterozygous, homozygous and bi-allelic) occurred in the *AtRPS4* single mutants (Table 2). The results demonstrate that multiple independent mutations can be obtained from just a single T1 line, which could be useful for generating allelic series.

**TABLE 2:** The representative mutations induced by AtEC1.2e1.1p:Cas9 in T2 plants of #18.

	*AtRPS4*		*AtRPS4B*	
WS2	**CCT**CTAGATGTACTGCTGAAGAG	WT	GTGATGCTTTTAGAGATCTTG**AGG**	WT
AtEC #18-1	CCTCTA**g**GATGTACTGCTGAAGAG	+1	GTGATGCTTTTAGAGATCTTGAGG	WT
	CCTCTA**g**GATGTACTGCTGAAGAG	+1	GTGATGCTTTTAGAGATCTTGAGG	WT
AtEC #18-2	CCTCTA–TGTACTGCTGAAGAG	−2	GTGATGCTTTTAGAGATCTTGAGG	WT
	CCTCTAGATGTACTGCTGAAGAG	WT	GTGATGCTTTTAGAGATCTTGAGG	WT
AtEC #18-3	CCTCTA-ATGTACTGCTGAAGAG	−1	GTGATGCTTTTAGAGATCTTGAGG	WT
	CCTCTA-ATGTACTGCTGAAGAG	−1	GTGATGCTTTTAGAGATCTTGAGG	WT
AtEC #18-4	CCTCTA**a**GATGTACTGCTGAAGAG	+1	GTGATGCTTTTAGAGATCTTGAGG	WT
	CCTCTAGATGTACTGCTGAAGAG	WT	GTGATGCTTTTAGAGATCTTGAGG	WT
AtEC #18-5	CCTCTA**a**GATGTACTGCTGAAGAG	+1	GTGATGCTTTTAGAGATCTTGAGG	WT
	CCTCTA-ATGTACTGCTGAAGAG	−1	GTGATGCTTTTAGAGATCTTGAGG	WT
AtEC #18-6	CCTCTA-ATGTACTGCTGAAGAG	−1	GTGATGCTTTTAGAGAT–TGAGG	−2
	CCTCTAGATGTACTGCTGAAGAG	WT	GTGATGCTTTTAGAGATC**t**TTGAGG	+1
AtEC #18-7	CCTCTA**c**GATGTACTGCTGAAGAG	+1	GTGATGCTTTTAGAGATCTTGAGG	WT
	CCTCTA**g**GATGTACTGCTGAAGAG	+1	GTGATGCTTTTAGAGAT-TTGAGG	−1

*The green letters are PAM sequences of Cas9 target sites. Lower letters are insertions and dashed lines are deletions induced by Cas9.*

**FIGURE 6:**
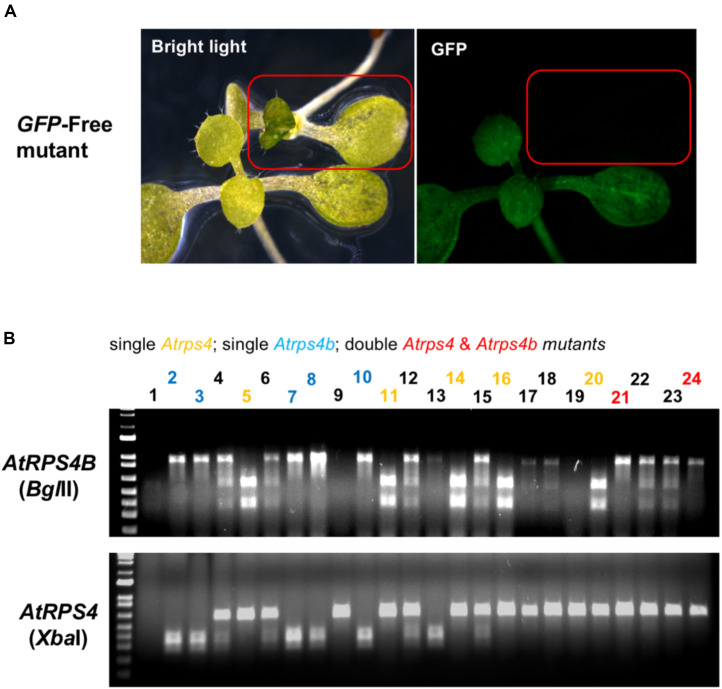
Selection of transgene-free, mutant Arabidopsis plants by absence of GFP fluorescence and PCR-RE genotyping. **(A)** T3 T-DNA-free edited plants can be selected under the fluorescence microscope. **(B)** Gel electrophoresis images of PCR-amplicons digested with restriction enzymes (*Bgl*II for *AtRPS4B* and *Xba*I for *AtRPS4*). Homozygous single and double mutants were obtained in the T3 generation.

The GFP fluorescence and PCR-RE approach were also used to select transgene-free mutants in the T3 generation (Figure 6). Through genetic segregation, non-GFP (transgene-free) T3 plants could be recovered that carried mutations in either *AtRPS4*, *AtRPS4B* or both genes. These results suggest that it is feasible to use visible GFP fluorescence to remove the transgenic plants, then use the PCR-RE approach to identify the mutants from the previous generation through genetic segregation. This strategy can be used to obtain transgene-free mutants from either the T2 or T3 generation.

### ECp:Cas9/gRNA Constructs Induce Mutagenesis of Two *GmAGO7* Genes in Soybean

To test whether our ECp-Cas9/gRNA systems could introduce mutations at genomic target loci in soybean, we chose *GmAGO7a* (Glyma.01G053100) and *GmAGO7b* (Glyma.02G111600) for targeted mutagenesis. In plants, ARGONAUTE (AGO) proteins are associated with small RNA (sRNA) mediated repression of gene expression through either direct cleavage or other mechanisms, such as target destabilization or translational repression (Carbonell et al., 2012; Meister, 2013). ARGONAUTE7 (AGO7), a key regulator in the trans-acting small interfering RNAs (ta-siRNA) pathway, plays a conserved role in controlling leaf pattern among species. In Arabidopsis, the *ago7* mutants display increased leaf length and downward-curled leaf margin due to accelerated juvenile-to-adult transition; however, the mutants did not show obvious defects in leaf polarity (Hunter et al., 2003, 2006; Fahlgren et al., 2006). Overexpressing *SlAGO7* in tomato exhibited pleiotropic phenotypes, including improved axillary bud formation, altered leaf morphology and inflorescence architecture, and increased fruit yield (Lin et al., 2016). Loss-of-function of *Mtago7* resulted in lobed leaf margins and more widely spaced lateral organs in *Medicago truncatula* (Zhou et al., 2013). There are two *AGO7* paralogous genes (*GmAGO7a* and *GmAGO7b*) in the annotated Williams 82 genome^2^.

**FIGURE 7:**
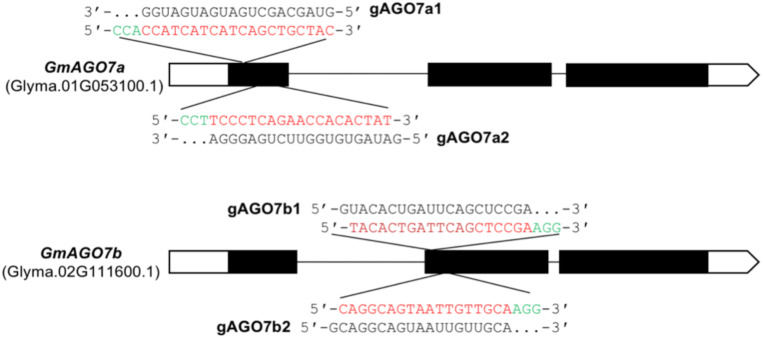
gRNA target sites in *GmAGO7a* and *GmAGO7b.* The structure of the *GmAGO7a* and *GmAGO7b* genes are represented by exons (black bars), introns (black lines) and UTRs (white bars). The sequences of the target sites are shown in red, PAM sequences in green and gRNA sequences in black. A total of four gRNAs were constructed into two Cas9 plasmids, one containing gAGO7a1 and gAGO7b2 (gAGO7a1/b2) and another containing gAGO7a2 and gAGO7b1 (gAGO7a2/b1).

We first verified the efficiency of the CRISPR-Cas9 system and activity of the gRNA in the soybean hairy root system. The CaMV double 35S (2x35S) promoter was used to drive expression of Cas9, and two gRNAs were designed to target a unique sequence in each gene. Cassettes expressing two different pairs of gRNAs targeting *GmAGO7a* and *GmAGO7b* – gAGO7a1 and gAGO7b2 (named gAGO7a1/b2) and gAGO7a2 and gAGO7b1 (named gAGO7a2/b1) – were constructed and each was recombined into the Cas9 binary vector, resulting in constructs that simultaneously targeted *GmAGO7a* and *GmAGO7b* (Figure 7).

The resulting two constructs were individually introduced into *Agrobacterium rhizogenes* strain K599 to induce hairy roots from infected soybean cotyledons. Hairy roots from individual cotyledons were collected and pooled for genomic DNA extraction. Individual DNA samples were used to PCR-amplify the relevant regions using the gene-specific primers for *GmAGO7a* and *GmAGO7b*. The T7 endonuclease I (T7E1) analysis, an assay that involves using T7E1 to digest the PCR amplicons of target genomic loci (Guschin et al., 2010), revealed a range of 80 to 100% mutation frequencies for gAGO7a1 and gAGO7a2, about 20% for gAGO7b1 and no detected mutation for gAGO7b2 (Supplementary Figure S2). The results indicated that the gRNAs (gAGO7a1 and gAGO7a2) for *GmAGO7a* and their promoters (U6-26 and U3b) were highly active, but the gAGO7b1 and gAGO7b2 gRNAs were less active.

**FIGURE 8:**
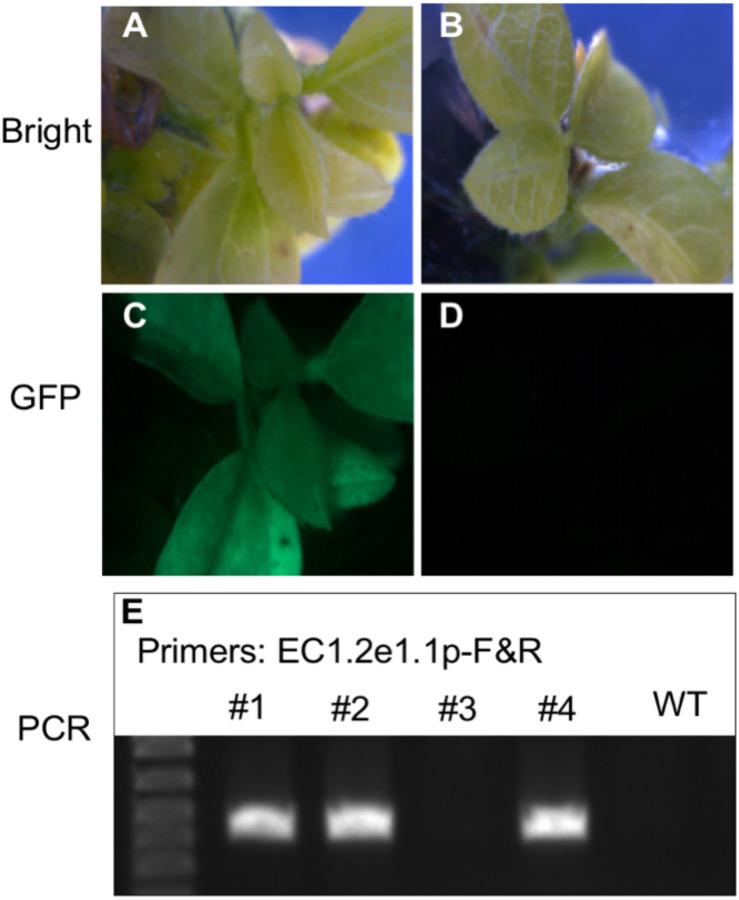
Detection of the transgenic soybean plants using GFP fluorescence and PCR-based genotyping. **(A–D)** Use of GFP fluorescence to detect transgenic soybean plants. **(A,C)** Images of a transgenic plant expressing GFP under bright and fluorescent light, respectively. **(B,D)** Images of a negative control plant under bright and fluorescent light. **(E)** Gel electrophoresis image of the PCR amplicons produced using T-DNA-specific primers targeting the promoter region.

To validate the ability of ECp:Cas9/gRNA to induce mutagenesis and examine the correlation of gRNA activities between hairy roots and stable transgenic plants, we used five constructs expressing Cas9 under different egg cell-specific promoters and gRNAs (gAGO7a2/b1 and gAGO7a1/b2) (pAtP5p:Cas9-gAGO7a1/b2, pAtP5p:Cas9-gAGO7a2/b1, pAtEC1.2e1.1p:Cas9-gAGO7a2/b1, pGmEC1.1p:Cas9-gAGO7a2/b1, and pGmEC1.2p:Cas9-gAGO7a2/b1) for stable soybean transformation. These five constructs were introduced individually into the Williams 82 cultivar of soybean by the *A. tumefaciens*-mediated transformation method. The GFP fluorescence was used to further screen the transgenic plants from the regenerated plants that survived from the herbicide glufosinate (Figures 8A–D). In addition, PCR-amplifications of Cas9 and gRNA genes were used to identify the transgenic plants (Figure 8E). Plants were screened by resistance to glufosinate (*bar*), GFP fluorescence and PCR-amplification, and 2 and 3 independent T0 transgenic plants were obtained for AtP5p:Cas9-gAGO7a1/b2 and AtP5p:Cas9-gAGO7a2/b1, respectively (Table 3). T0 plants were grown to maturity so that we could determine the mutation efficiency in the T1 generation. The three T0 AtP5p:Cas9+gGmAGO7a2/b1 lines (ST397-1, -4, and -5), produced transgenic progeny, but none that were tested carried a mutation at either gRNA target sites. As expected, the T0 AtP5p:Cas9-gAGO7a2/b1 plants (ST397-2 and -3) that survived Bar selection but were negative for GFP fluorescence and the PCR tests for the Cas9 and gRNA transgenes produced no transgenic progeny and carried no mutations at the *GmAGO7a* or *GmAGO7b* target sites (Table 3).

**TABLE 3:** Genotyping analysis of T0 and T1 transgenic soybean plants from five CRISPR constructs.

		T0				T1	
CRISPR Construct	Line	*Bar*	*GFP*	*Cas9*	gRNA	Transgenic vs. (Total plants)	# of mutant in *GmAGO7a*
AtP5p:Cas9 (gAGO7a1/b2)	ST398-4	+	+	+	+	0(19)	None
	ST398-5	+	+	+	+	0(27)	None
AtP5p:Cas9 (gAGO7a2/b1)	ST397-1	+	+	+	+	11(26)	None
	ST397-2	+	−	−	−	0(27)	None
	ST397-3	+	−	−	−	0(25)	None
	ST397-4	+	+	+	+	11(27)	None
	ST397-5	+	+	+	+	17(27)	None
	ST397-10	+	−	−	−	T0 died	
AtEC1.2e1.1p:Cas9 (gAGO7a2/b1)	ST410-4	+	ND	+	+	6(15)	2
	ST410-7	+	ND	+	+	1(18)	None
	ST410-8	+	ND	+	+	5(15)	None
	ST410-9	+	ND	-	-	T0 died	
	ST410-11	+	ND	+	+	0(17)	None
	ST410-13	+	ND	+	+	2(2)	None
	ST410-14	+	ND	+	+	21(23)	7
	ST410-18	+	ND	+	+	3(3)	None
	ST410-25	+	ND	+	+	30(36)	4
	ST410-32	+	ND	+	+	11(15)	None
	ST410-35	+	ND	+	+	0(14)	None
	ST410-36	+	ND	+	+	12(12)	7
	ST410-40	+	ND	+	+	0(17)	None
	ST410-44	+	ND	+	+	6(6)	6
GmEC1.1p:Cas9 (gAGO7a2/b1)	ST411-6	+	ND	−	−	0(6)	None
	ST411-12	+	ND	+	+	8(18)	None
	ST411-17	+	ND	−	−	0(9)	None
	ST411-18	+	ND	+	+	4(11)	None
GmEC1.2p:Cas9 (gAGO7a2/b1)	ST412-20	+	ND	+	+	T0 died	
	ST412-29	+	ND	−	−	T0 died	
	ST412-27	+	ND	−	−	T0 died	
	ST412-31	+	ND	−	−	0(14)	None

*ND, no GFP detection was performed.*

For the AtEC1.2e1.1p, GmEC1.1p and GmEC1.2p constructs, 13, 2, and 1 independent T0 transgenic lines were positive for glufosinate screen (*bar*) and the PCR amplification, respectively (Table 3). Out of the 13 T0 lines from the AtEC1.2e1.1p:Cas9-gAGO7a2/b1 construct, 10 lines produced transgenic T1 progeny, and of these, five lines produced T1 progeny some of which carried mutations in *GmAGO7a*, but none had mutations in *GmAGO7b*. Among the 97 T1 progeny plants from the five lines, 26 plants contained mutations for *GmAGO7a* a mutation efficiency of 26.8% based on the T7E1 assay (Table 3). For other non-transgenic plants, we also checked for occurrence of the mutation at both target loci, and as expected no mutation was found in these plants. For the GmEC1.1p:Cas9-gAGO7a2/b1, two transgenic lines (ST411-12, -18) produced transgenic progeny, but no mutation was detected at either target site. Unfortunately, no seed was produced from the only transgenic line derived from GmEC1.2p:Cas9-gAGO7a2/b1. These results indicate that the AtEC1.2e1.1p promoter is likely the most efficient for gene editing in soybean and that the frequencies of mutations induced by the gRNA were consistent with the hairy root system.

**FIGURE 9:**
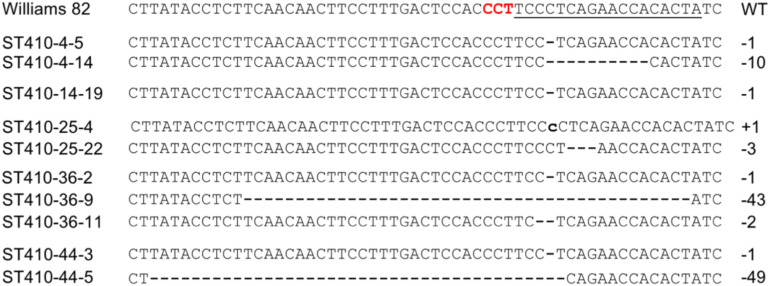
Representative mutations induced by egg cell-specific expression of Cas9 in soybean plants derived from five independent T0 lines. Red letters and underlined letters in Williams 82 indicate the respective PAM and gRNA target sequences. Dashed lines and lowercase letters denote deletions and insertions, respectively, at the *GmAGO7a* target site.

We expected that Cas9 would be activated in egg cells, zygotes or early embryos beyond the T1 generation. Therefore, we further examined the prevalence of mutations in T2 plants produced from T1 lines that were derived from AtEC1.2e1.1p:Cas9-gAGO7a2/b1 and carried the Cas9 and gRNA transgenes. A total of 31 T1 lines produced from 6 T0 lines were selected for further analysis in the T2 generation. Twenty-two T1 lines produced T2 plants that carried inheritable transgenes and mutations, while seven T1 lines (ST410-14-19, ST410-25-22, ST410-36-2, ST410-36-4, ST410-36-9, ST410-36-11, ST410-44-6) did not produce any seed (Table 4). A total of 145 out of 389 T2 plants tested positive for mutations in *GmAGO7a* by using the T7E1 assay. Sanger sequencing the PCR-amplicons from some of the T7E1 positive plants revealed site-specific mutations in *GmAGO7a* (Figure 9). Most mutants were heterozygous, but 11 were homozygous from T1 lines ST410-4-5, ST410-4-14, ST410-25-4, ST410-44-5) (Table 4). We found only one mutant for *GmAGO7b* among 389 T2 plants (ST410-14-3-7) (Table 4). We were unable to correlate a mutant phenotype with homozygous mutations in *GmAGO7a*, suggesting that there is a redundant function provided by *GmAGO7b.* The results indicate that Cas9 and gRNAs are still active in egg cells, zygotes and early embryos in the T1 and T2 generations. For construct GmEC1.1p:Cas9-gAGO7a2/b1, eight T1 lines were selected for analyses of transgene presence and occurrence of mutations in the T2 generation. No mutation was detected in either *GmAGO7a* or *GmAGO7b* among 140 T2 plants (Table 4). These results demonstrated that the AtEC1.2e1.1p promoter is the best promoter for egg cell-specific Cas9 expression and genome editing in Arabidopsis and soybean.

**TABLE 4:** Genotyping analysis of CRISPR/Cas9-induced mutations in T1 and T2 soybean plants.

CRISPR construct	T0 Line	T1		T2		
		No.	Mutation (*GmAGO7a*)	Mutant plants (*GmAGO7a*)	Mutant plants (*GmAGO7b*)	Total plants
AtEC1.2e1.1p:Cas9 (gAGO7a2/b1)	ST410-4	#3	−	0	0	21
		#4	−	0	0	18
		#5	+	14 (3 Homo-)	0	20
		#6	−	0	0	19
		#14	+	11 (2 Homo-)	0	20
		#15	−	0	0	15
	ST410-14	#2	+	7	0	16
		#3	+	0	1	15
		#6	+	9	0	15
		#19	+	ND	ND	ND
	ST410-25	#3	+	7	0	16
		#4	+	9 (2 Homo-)	0	14
		#8	+	8	0	13
		#22	+	ND	ND	ND
	ST410-32	#1	−	0	0	17
		#2	−	0	0	20
		#5	−	0	0	17
		#12	−	0	0	18
	ST410-36	#1	+	1	0	14
		#2	+	ND	ND	ND
		#3	+	4	0	16
		#4	−	ND	ND	ND
		#6	+	9	0	19
		#9	+	ND	ND	ND
		#11	+	ND	ND	ND
	ST410-44	#1	+	8	0	8
		#2	+	9	0	9
		#3	+	10	0	10
		#4	+	19	0	19
		#5	+	20 (4 Homo-)	0	20
		#6	+	ND	ND	ND
**Sum**	**6**	**31**	**22**	**145**	**1**	**389**
GmEC1.1p:Cas9 (gAGO7a2/b1)	ST411-12	#2	−	0	0	19
		#5	−	0	0	17
		#17	−	0	0	18
		#18	−	0	0	18
	ST411-18	#6	−	0	0	18
		#12	−	0	0	18
		#14	−	0	0	18
		#20	−	0	0	14
**Sum**	**2**	**8**	**0**	**0**	**0**	**140**

*ND, no seeds or DNA samples were collected.*

## Discussion

In the present study, we built a CRISPR/Cas9 platform for genome editing in soybean with the aid of proof-of-concept experiments in Arabidopsis. The system depends on the conditional expression of Cas9 at the reproductive stage by using an egg cell-specific promoter. The use of tissue-specific promoters is expected to have multiple benefits that includes reducing the potential toxicity associated with Cas9 expressed under the otherwise strong and constitutive promoters. In addition, the expression of Cas9 in germ cells (egg cells, zygotes, and early embryos) results in heritable edits and reduces somatic mutations in plants derived from organogenesis, which has proven to be a feasible approach particularly in Arabidopsis (Wang et al., 2015; Yan et al., 2015; Mao et al., 2016). Our CRISPR system also features a visual marker (GFP) under a soybean ubiquitin gene promoter that can be used to avoid growing and maintaining escapes from soybean transformation projects. The system can be used for multiplex genome editing as up to 6 gRNA cassettes can be integrated into a single construct. Finally, we validated the system for site-specific mutagenesis by targeting two genes each in soybean and Arabidopsis up to the T2 and T3 generations, respectively.

Genome editing in soybean still faces several challenges although numerous studies have demonstrated its feasibility. First, efficient and reliable transformation technology in soybean has lagged behind some other crop species. Genetic transformation of soybean is still genotype dependent, and only a very limited number of genotypes are transformable (Yamada et al., 2012; Altpeter et al., 2016). The most widely and routinely used transformation platforms depend on organogenesis, a process that is based on cotyledonary-nodes as explants and *Agrobacterium*-mediated DNA delivery, to regenerate whole plants (Paz et al., 2006). This method often leads to a high occurrence of escape and chimeric plants, and it makes soybean transformation labor-intensive and expensive. The number of genuine transgenic plants is usually low from each transformation project. We observed evidence for escapes and chimerism during analyses of T0 and T1 plants in which some T0 plants that were Bar positive surviving glufosinate selection, but then tested negative for GFP and/or the Cas9 and gRNA transgenes (Table 3). In addition, some T0 lines that tested positive for Bar, GFP, and the Cas9 and gRNA transgenes were likely chimeric, because their progeny neither inherited the transgenes nor carried mutations in the target genes (Table 3). Our soybean CRISPR/Cas9 system, therefore, is ideal, because it allows us to select the Cas9/gRNA positive plants that can be used to generate many progenies that each contain independent and inheritable mutated alleles.

We tested four different EC promoters and found only one (AtEC1.2e1.1p) was capable of inducing mutations in progenies of T1 and T2 generations in soybean. There are possible explanations for the failure of the other three promoters. First, Cas9 was expressed but not as levels sufficient to cause DSBs within the context of chromatin during the reproductive stage of soybean plants. It is worth to point out that two promoters (AtP5p and GmEC1.1p) are expected to be active, because they did enable Cas9 to induce mutagenesis in Arabidopsis. However, the third promoter (GmEC1.2p) may not be functional, because Cas9 failed to induce mutagenesis in both Arabidopsis and soybean. Future work is needed to determine whether Cas9 mRNA and protein expression is activated by the three promoters. Second, the guide RNAs used in the present study may not be the most active ones. More guide RNAs remain to be designed and tested. Finally, the number of T0 transgenic soybean plants derived from each of the three constructs was small, which is a common limitation of soybean transformation. We obtained only eight, four and four T0 plants from AtP5p:Cas9, GmEC1.1p:Cas9, and Gm1.2p:Cas9, respectively, while 14 T0 plants were generated from AtEC1.2e1.1p:Cas9. The low number of T0 plants prevents us from making definitive conclusions about the three promoters that did not induce mutations in soybean. Future work to increase the efficiency of soybean transformation will make it possible to generate larger numbers of T0 plants with constructs carrying more gRNA and Cas9 configurations.

In the present study, we tested the correlation of gRNA activities among hairy roots and stable transgenic plants. Hairy roots yielded very high frequencies of mutagenesis for two of four gRNAs (gAGO7a1 and gAGO7a2) and relatively low or no activity by another two (gAGO7b1 and gAGO7b2). Similarly, the frequency of mutations induced by gAGO7a2 is much higher than gAGO7b1 in the stable transgenic plants. The findings suggest that the hairy root system is a quick way to test and select highly active gRNA before proceeding to stable transformation.

The second challenge facing genome editing in soybean is the genome size and complexity. Soybean, e.g., Williams 82, is a palaeopolyploid and contains a genome of ∼1.15 gigabases (Schmutz et al., 2010), about eight times as larger than the *A. thaliana* genome (135 megabases) (Initiative, 2000). Prior to site-specific cleavage of the target DNA, Cas9 and gRNA first recognize the PAM sequence and then the PAM-proximal region through the complementarity between the gRNA and target strand (Sternberg et al., 2014). The implication of this mode of recognition is that the genome editing efficiency is expected to be negatively correlated to the genome size and complexity. Our data from Arabidopsis and soybean supports this notion. For instance, three of the egg cell-specific promoters driving Cas9 could produce mutants in Arabidopsis, while only one of three was able to do so in soybean at much lower percentage. Alternatively, the expression levels of Cas9 and gRNA may be higher in Arabidopsis than in soybean.

It is highly likely the editing efficiency would be correlated with the abundance of Cas9/gRNA ribonucleoprotein complexes. Based on this, to further improve our soybean CRISPR/Cas9 system, several strategies and approaches can be explored. First, we are interested in approaches to increase the abundance of Cas9 in egg cells, zygotes and early embryos of the T0 or T1 generation transgenic plants. For example, it was shown that zCas9 (maize codon-optimized Cas9) coding sequence followed by the pea ribulose-1,5-bisphosphate carboxylase small subunit (*rbcS*) E9 gene terminator (rbcS E9t) induced edits at higher rates than zCas9 followed by the *Agrobacterium Nos* gene terminator in Arabidopsis (Wang et al., 2015). The constructs described in the present study all carried Cas9 followed by the *Nos* terminator. In addition, the incorporation of introns in gene coding regions especially at the 5′ end was shown to enhance gene expression (Le Hir et al., 2003). Therefore, use of a different terminator (e.g., pea rbcS E9 or soybean rbcS1 terminator) along with soybean codon-optimized Cas9+intron could increase expression level of Cas9 transcripts expressed under control of the EC promoters. It may also be possible to use soybean U6 promoters for expressing gRNAs to improve the genome editing efficiency (Di et al., 2019). Such modifications to increase the levels of activated Cas9/gRNA complexes in germline cells are expected to increase the frequency of targeted mutagenesis without sacrificing germline-specific expression.

## Materials and Methods

### Plant Materials

*Arabidopsis thaliana* (ecotypes Wassilewskija-2, Ws-2) plants were grown vertically on half-strength Murashige and Skoog (1/2 MS) plates at pH 5.6–5.8 (adjusted with 1 N KOH), supplemented with 0.85% (*w*/*v*) agar and 1% (*w*/*v*) sucrose. All plants were grown at 22°C under long-day conditions (16 h light/8 h dark).

The soybean cultivar Williams 82 was used for hairy root transformation and whole plant stable transformation. Soybean seeds were surface-sterilized for 14–16 h with chlorine gas. Seeds were germinated on 1/4 Gamborg’s solid medium under long-day conditions (16-h light/8-h dark, 28°C/24°C) in growth chambers. After 5 days, healthy plants were selected for hairy root transformation. Soybean plants were grown in clay pots containing Pro-mix Bx Biofungicide potting mix supplemented with Osmocote slow-releasing fertilizer (14-14-14) and were grown in greenhouse to maturity under a photoperiod of 16 h light and 8 h dark at 28–24°C.

### Vector Construction

Different ECp were used to drive Cas9 expression in this study. The Arabidopsis AtP5 promoter (919 bp, Chr1:26929821…26930831 in TAIR 10) fused to Cas9 (pCambia:AtP5p:Cas9) was constructed in pCAMBIA3300. This vector was modified by inserting the cassette of *att*R1-ccdB-*att*R2, which is used to insert gRNA modules by Gateway recombination, resulting in the destination vector, pGW-AtP5p:Cas9. The AtEC1.2e1.1p ECp (1,362 bp, Chr2:9282423…9283302 + Chr1:28810535…28811064 in TAIR 10) was assembled through overlapping PCR amplification using Arabidopsis genomic DNA and primers. To identify soybean ECp, the Arabidopsis egg cell secreted protein 1.1 (AT1G76750) was used as a query to BLAST search against the soybean reference genome to retrieve seven egg cell secreted protein sequences and their promoter sequences. Promoters of GmEC1.1 (1,446 bp, Chr20:40598179…40599767 in Wm82.a2v1) and GmEC1.2 (1,334 bp, Chr06:18296898…18298365 in Wm82.a2v1) were PCR-amplified using soybean genomic DNA as a template and gene-specific primers. GmEC1.1 and GmEC1.2 each represent one member of their clades among the seven GmECs (Supplementary Figure S3). To construct a Cas9 expression cassette with the 2x35S promoter, the 2x35S promoter was PCR-amplified from the pTF101 plasmid. All the promoters were swapped with the AtP5p in pCambia:AtP5p:Cas9 at *Nco*I and *Spe*I through the Gibson cloning method, resulting in constructs containing each of the four different promoters for expression of Cas9. The GmUbi-GFP-NosT cassette was then inserted into each vector through restriction and ligation at the *Hind*III site using the standard molecular cloning methods (Ausubel et al., 1998), resulting in the GFP version of the plasmids. The primer information is provided in Supplementary Table S4.

For the construction of gRNA genes, the intermediate vectors pENTR4-ccdB which was modified by inserting the cassette of *att*L1-ccdB-*att*L2. Briefly, each gRNA vector (pCRgRNA) has a unique promoter and poly-T terminator. The specific gRNA spacer sequence was inserted at the two *Bsm*BI restrict sites. To construct a specific gRNA gene, two 20 – 24 nt complementary oligonucleotides were annealed to produce a double-stranded DNA oligonucleotide (dsOligo). To make a 2-gRNAs cassette, the first dsOligo was designed with a 5′ overhang of ATTG in the sense strand and a 5′ of overhang AAAC in the antisense strand; and the second dsOligo was designed to contain a 5′ overhang of GTCA in the sense strand and a 5′ of overhang AAAC in the antisense strand. All oligonucleotides were synthesized and purchased from Integrated DNA Technology (Coralville, IA, United States). The individual gRNA cassettes were assembled into the pENTR4-ccdB vector by the Golden Gate assembly method using *Bsa*I. After sequencing the guide RNA regions, the gRNA cassette was finally mobilized to four different pGW-ECp:Cas9-GFP-ccdB constructs by using Gateway LR Clonase (Thermo Fisher Scientific, Waltham, MA, United States).

*Escherichia coli* strain DH5α and DB3.1 were used for molecular cloning of Cas9/gRNA constructs. *Agrobacterium tumefaciens* strain GV3101 and EHA105 were used for Arabidopsis and soybean transformation, respectively. *A. rhizogenes* strain K599 was used for the soybean hairy root transformation. *E. coli* cells were grown in Luria–Bertani (LB) medium supplemented with appropriate antibiotics at 37°C with a standard culture technique, while *Agrobacterium* strains were grown at 28°C in Luria–Bertani (LB) medium with appropriate antibiotics (Ausubel et al., 1998).

### Hairy Root Transformation

*Agrobacterium rhizogenes* strain K599 containing the Cas9/gRNA binary constructs was used for the soybean hairy root induction. Soybean cotyledons of cultivar Williams 82 were inoculated with the transformed K599 strain using a previously described protocol (Kereszt et al., 2007) with slight modifications. Bacterial cells were scraped from the plates and suspended in 1 mL sterile water. The blades of sterilized scissors were immersed into the bacterial suspension, then used to cut off the 1/4 of the cotyledon that was attached to the stem. The cut cotyledons were placed on a stack of one or two sterile pre-wet paper towels (with 1/4 Gamborg’s liquid medium plus 200 μg/ml Timentin) in an ice-cream box. The cotyledons were kept in a growth chamber with a photoperiod of 16-h light/8-h dark at 28°C/24°C. After cultivation for 2∼3 weeks, hairy roots were collected for further analysis. Hairy roots induced by K599 lacking CRISPR construct were used as control.

### Transformation of Arabidopsis and Soybean

Arabidopsis transformation was performed by using the floral dipping protocol as described (Pike et al., 2019). *Agrobacterium*-mediated transformation of a fixed number of half seed explants of Williams 82 genotype was performed at the Iowa State University Plant Transformation Facility as described (Paz et al., 2004). The plants were grown in greenhouses with a 16-h day at 28°C and an 8-h night at 24°C. The transgenic plants were further confirmed by GFP fluorescence and PCR assays on the Cas9 and gRNA transgenes.

### Molecular Characterization of CRISPR Plants of Arabidopsis and Soybean

Genomic DNA samples were extracted from Arabidopsis leaves using the CTAB (cetyltrimethyl ammonium bromide) method (Murray and Thompson, 1980). Soybean genomic DNA was extracted from newly expanding primary leaves of T1 and T2 seedlings using the QIAamp Fast DNA Tissue Kit (Qiagen, Germantown, MD, United States). Genomic DNA was used for PCR-amplification of relevant regions with specific primers flanking the target sites (Supplementary Table S4). PCR reaction conditions were optimized for each primer pair and are available upon request. PCR amplicons were assessed for mutations using the T7 endonuclease I (T7E1) assay or restrict enzyme digestion and Sanger sequencing. For the T7E1 assay, PCR-amplicons obtained from the transgenic tissues were mixed with the respective amplicon derived from wild type, denatured (95°C for 5 min) and reannealed (ramp down to 25°C at 5°C/min), then subjected to T7E1. For PCR-RE, the PCR-amplicons were used directly for digestion with appropriate restriction enzymes. For sequencing, the PCR amplicons derived from the T7E1-positive samples were treated with ExoSAP-IT (Affymetrix, Santa Clara, CA, United States) and subsequently evaluated by the Sanger sequencing method by the University of Missouri-Columbia DNA Core Facility^3^. The sequencing chromatograms were carefully examined for exact patterns that might indicate mono-allelic or bi-allelic mutations.

## Data Availability Statement

All datasets generated for this study are included in the article/Supplementary Material.

## Author Contributions

BY, SW, and SL designed the research. NZ, TL, JD, JS, and RL performed the experiments. DP and WG analyzed the data. NZ and BY wrote the manuscript with input from all other authors.

## Conflict of Interest

The authors declare that the research was conducted in the absence of any commercial or financial relationships that could be construed as a potential conflict of interest.

## Footnotes

1 http://cbc.gdcb.iastate.edu/cgat/

2 phytozome.jgi.doe.gov

3 https://dnacore.missouri.edu
