# Supplementary material for: CRISPR/Cas9-Based Gene Editing Using Egg Cell-Specific Promoters in Arabidopsis and Soybean
Source: Front Plant Sci. 2020 Jun 16;11:800. doi: 10.3389/fpls.2020.00800 (PMC7309964; doi:10.3389/fpls.2020.00800)
Supplement: Supplementary file 1 [file Data_Sheet_1.pdf]

CRISPR/Cas9-Based Gene Editing Using Egg Cell-Specific Promoters in Arabidopsis and Soybean

Na Zheng[1,2], Ting Li[3], Jaime D. Dittman[4], Jianbin Su[2], Riqing Li[2], Walter Gassmann[2], Deliang Peng[1], Steven A. Whitham[4,*], Shiming Liu[1,*] and Bing Yang[2,5,*]

Supplementary Material

Table S1. Information about plasmids for egg cell-specific CRISPR/Cas9 system

Table S2. Genotypes of T1 plants derived from four different CRISPR constructs in Arabidopsis

Table S3. Segregation of markers of T2 plants from T1 lines in Arabidopsis

Table S4. Oligonucleotides used in this study

Figure S1 Map of four ECp vectors.

Figure S2 Detection of targeted mutations in *GmAGO7a* and *GmAGO7b* in soybean hairy roots.

Figure S3 Phylogram of nine EC secreted proteins from Arabidopsis and soybean.

Table S1. Information about plasmids for egg-cell specific CRISPR/Cas9 system

Plasmid		Overhang after *BsmB*I Digestion		Overhang after *Bsa*I Digestion		Anti-biotics	Plasmid Size
		5′ end (5′- 3′)	3′ end (5′-3′)	5′ end (5′-3′)	3′ end (5′-3′)		
Modular units	pCRgRNA1	ATTG	GTTT	GCTT	CTGA	Spe^R	~3,500 bp
	pCRgRNA2	GTCA	GTTT	CTGA	AAGA		
	pCRgRNA2T				TAGC		
	pCRgRNA3	ATTG	GTTT	AAGA	GACT		
	pCRgRNA4	GTCA	GTTT	GACT	CGGT		
	pCRgRNA4T				TAGC		
	pCRgRNA5	ATTG	GTTT	CGGT	CTAT		
	pCRgRNA6	GTCA	GTTT	CTAT	TAGC		
Intermediate plasmid	pENTR-ccdB	NA	NA	GCTT	TAGC	Kan^R	4,092 bp
Destination plasmid	pGW-AtP5p:Cas9 -GmUbi:GFP	NA	NA	NA	NA		17,953 bp
	pGW-AtEC1.2e1.1p:Cas9 - GmUbi:GFP	NA	NA	NA	NA		18,379 bp
	pGW-GmEC1.1p:Cas9-GmUbi:GFP	NA	NA	NA	NA		18,476 bp
	pGW-GmEC1.2p:Cas9-GmUbi:GFP	NA	NA	NA	NA		18,365 bp

Table S2. Genotypes of T1 lines derived from four different CRISPR constructs in Arabidopsis

**CRISPR construct**	**Basta (+)**	**GFP (+)**	**PCR of Cas9/ gRNA (+)**	**Mutant** (+)	**Efficiency**
AtP5p:Cas9-gRNA	2	2	2	0	0
AtEC1.2e1.1p:Cas9-gRNA	25	20	20	3	15%
GmEC1.1p:Cas9-gRNA	35	28	28	0	0
GmEC1.2p:Cas9-gRNA	5	4	4	0	0

Table S3. Segregation of markers of T2 plants from T1 lines in Arabidopsis

**CRISPR construct**	**Total T1 lines analyzed**	**Basta selection**		**Green fluorescence detection**		**# T1 lines with *Bar* segregating at 3:1**
		**# T1 lines with some T2 plants survived**	**# T1 lines with all T2 plants died**	**# T1 lines producing fluorescent T2 plants**	**# T1 lines not producing fluorescent T2 plants**	
AtP5p:Cas9-gRNA	50	45	5	40	10	29
AtEC1.2e1.1p:Cas9-gRNA	78	76	2	68	10	54
GmEC1.1p:Cas9-gRNA	100	98	2	80	20	65
GmEC1.2p:Cas9-gRNA	5	5	0	4	1	0

Table S4. Oligonucleotides used in this study

Oligo name	Sequence (5′ to 3′)	Usage
gAtRPS4-F	gtcaTCTTCAGCAGTACATCTAG	dsOligo to construct gAtRPS4
gAtRPS4-R	aaacCTAGATGTACTGCTGAAGA	
gAtRPS4B-F	attgATGCTTTTAGAGATCTTG	dsOligo to construct gAtRPS4B
gAtRPS4B-R	aaacCAAGATCTCTAAAAGCAT	
AtRPS4-F	TCTAACGCACACCAGTGAGGA	PCR-amplification of *AtRPS4*
AtRPS4-R	CTTGTCAATGATGATGCCCAT	
AtRPS4B-F	TCGTCAGCCATCTCGTAGAAG	PCR-amplification of *AtRPS4*B
AtRPS4B-R	TTCGCCTCCTACCGAACTTAG	
gGmAGO7a1F1	attgTAGCAGCTGATGATGATGG	dsOligo to construct gGmAGO7a1
gGmAGO7a1R1	aaacCCATCATCATCAGCTGCTA	
gGmAGO7b1F1	attgTACACTGATTCAGCTCCGA	dsOligo to construct gGmAGO7b1
gGmAGO7b1R1	aaacTCGGAGCTGAATCAGTGTA	
gGmAGO7a2F1	gtcaTAGTGTGGTTCTGAGGGA	dsOligo to construct gGmAGO7a2
gGmAGO7a2R1	aaacTCCCTCAGAACCACACTA	
gGmAGO7b2F1	gtcaGGCAGTAATTGTTGCAA	dsOligo to construct gGmAGO7b2
gGmAGO7b2R1	aaacTTGCAACAATTACTGCC	
GmAGO7a-F	CATGGAAGAGACAGATGAG	PCR-amplification of *GmAGO7a*
GmAGO7a-R	ACCAGGATTAAGTGGTAGT	
GmAGO7b-F	GTAACTTGAGCTTACCATACTG	PCR-amplification of *GmAGO7b*
GmAGO7b-R	CATATGCTGGAGTAGCACCAC	
AtEC-F1	ATGAGATAAACCAATAACTAGCCATGGAATAA AAGCATTTGCGTTTG	PCR-amplify the AtEC1.1 enhancer
AtEC-R1	CTAATTCATGATAGGCGTTAGCTTAGTGGTGAT TTAAG	
AtEC-F2	CTTAAATCACCACTAAGCTAACGCCTATCATGA ATTAG	PCR-amplify the AtEC1.1 promoter
AtEC-R2	TGTTGTAAAAATACCGATGACTAGTATTTCTCA ACAGATTGATAAG	
GmEC1.1F1	ATGAGATAAACCAATAACTAGccatggCTAGTATG ATCCTTGCTAC	PCR-amplify the GmEC1.1 promoter
GmEC1.1R1	TGTTGTAAAAATACCGATGActagtATTCTTATAG AATATGCATATGC	
GmEC1.2F1	ATGAGATAAACCAATAACTAGccatggTAGTAATC GATTACATC	PCR-amplify the GmEC1.2 promoter

GmEC1.2R1	TGTTGTAAAAATACCGATGActagtATAGATGCTG ATGACATCAATG	
pCR8-R	TGTTGTGGTGTGTAGGGACAG	Sequence the gRNA genes
EC1.2e1.1p-F	TCGACCTTATCAATCTGTTGAG	Detect egg cell promoter for Cas9 gene via PCR
EC1.2e1.1p-R	CTGCAGTCTCCCCACTATCGAAAAG	
Cas9-F	TTGGGCAGTCATTACAGACG	Detect Cas9 gene via PCR
Cas9-R	CCTTGGCCATTTCGTTAGAG	

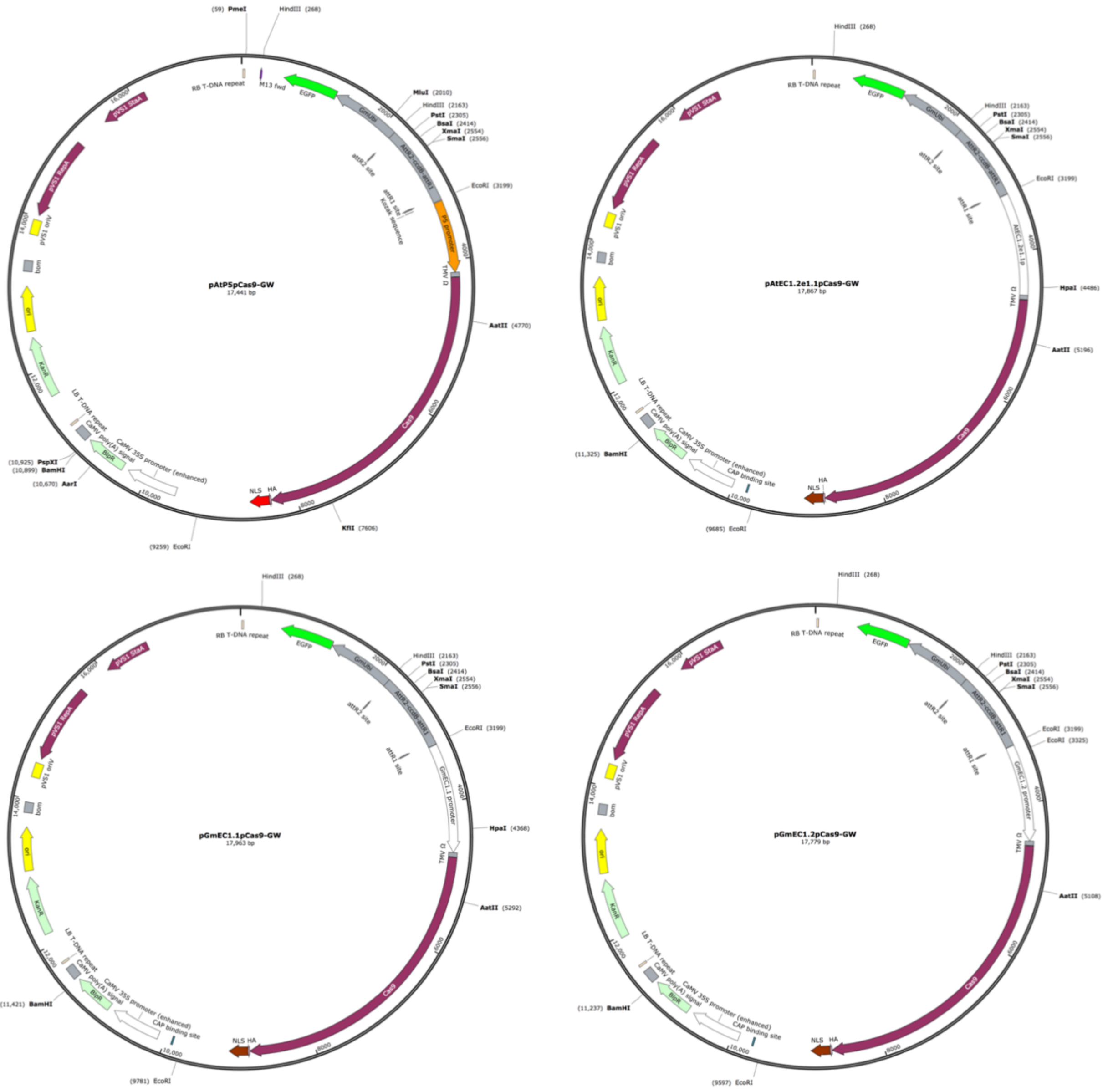

Figure S1 Map of four ECp vectors.

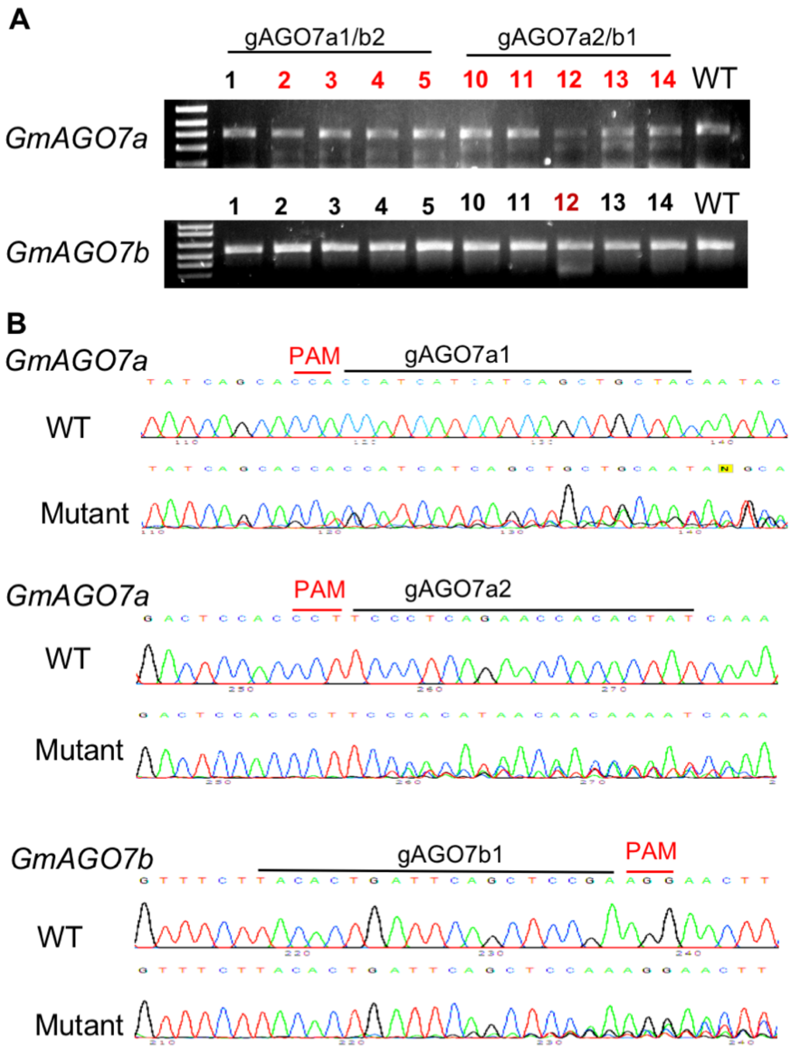

Figure S2 Detection of targeted mutations in *GmAGO7a* and *GmAGO7b* in soybean hairy roots. A. DNA gel electrophoresis images of PCR products after digestion with T7 endonuclease I (T7E1 assay). B. Sequencing chromatograms of PCR-amplicons derived from wildtype (WT) and mutagenized (Mutant) target sites.

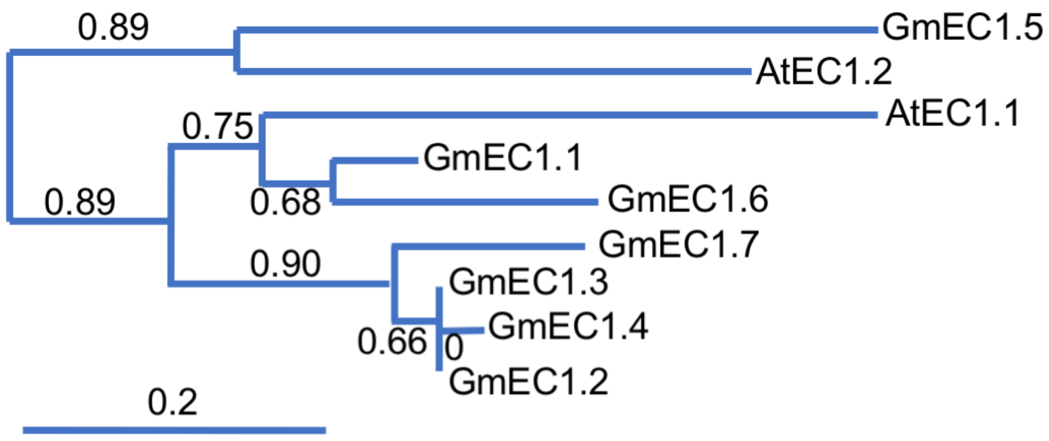

Figure S3 Phylogram of nine EC secreted proteins from Arabidopsis and soybean. The phylogram was by reconstructed using the maximum likelihood method in PhyML (www.phylogeny.fr) (Dereeper et al., 2008).

Dereeper, A., V. Guignon, G. Blanc, S. Audic, S. Buffet, F. Chevenet, J. F. Dufayard, S. Guindon, V. Lefort, M. Lescot, J. M. Claverie and O. Gascuel (2008). "Phylogeny.fr: robust phylogenetic analysis for the non-specialist." Nucleic Acids Res 36 (Web Server issue): W465-469.
